# Enhanced nonlinear characteristics with the assistance of a $\mathscr{PT}$-symmetric trimer system

**DOI:** 10.1038/s41598-018-21137-y

**Published:** 2018-02-13

**Authors:** Lei Du, Yan Zhang, Chu-Hui Fan, Yi-Mou Liu, Feng Gao, Jin-Hui Wu

**Affiliations:** 1 Center for Quantum Sciences and School of Physics, Northeast Normal University, Changchun, 130117 P. R. China; 2 College of Physics, Jilin University, Changchun, 130012 P. R. China

## Abstract

We study the parity-time (PT) symmetry characteristics and the applications to nonlinear optics in an optical trimer system consisting of two indirectly coupled standing-mode micro-cavities and a two-level quantum emitter (QE) placed at the intersection of two cavities. We find this trimer system can exhibit analogical phenomena as those in typical $\mathscr{PT}$-symmetric dimer systems composed of a passive cavity directly coupled to an active cavity. This system, whose $\mathscr{PT}$ symmetry is demonstrated by our analytic results, can be transformed between the $\mathscr{PT}$-symmetric phase and the $\mathscr{PT}$-broken phase by adjusting relevant system parameters. Then, with this system, we observe both the linear and nonlinear parts of the transmission field become remarkably enhanced and can further reach peak values around the $\mathscr{PT}$ breaking point. In addition, we show the negative correlation between the gain degree of the linear (nonlinear) transmission part and decay rate of the QE. This trimer proposal is feasible for experiments and may provide a promising platform for $\mathscr{PT}$-symmetric optics of low-light levels. Moreover, novel phenomena arising from the QE-cavity-coupling induced nonlinearity gain could be explored to fabricate photonic devices and controllable nonlinear optical media for quantum information process and communication of photons.

## Introduction

As a branch of modern optics, nonlinear optics expounds optical response properties in nonlinear media under the action of strong coherent lights and is attracting broad interests in view of its fundament features and numerous applications^1^, e.g. high-order sideband generation^2–4^, two-photon absorption^5–7^, optical parametric oscillation and amplification^8–10^, and optical Kerr effect^11–13^. As one of the most advantageous optical devices, optical microcavities possessing ultrahigh quality factors and fineness can hugely enhance the light-matter interactions so that many nonlinear phenomena can be obviously amplified. Thus, more recently, microcavity system has become an excellent platform for investigating nonlinear optics^14–20^ by means of quite a number of quantum effects, particularly $\mathscr{PT}$ symmetry effects^21–24^.

Since its conception first proposed by Bender *et al*. as a criterion for a non-Hermitian Hamiltonian with a real eigenvalue spectrum in quantum mechanics^25,26^, $\mathscr{PT}$ symmetry has rapidly spread out to plenty of physical fields, especially optics and photonics, due to several reasons, one of which is all-real linear spectra and the existence of nonlinear steady states with continuous ranges of energy values^27^. As is well-known, an unique feature of the $\mathscr{PT}$-symmetric optical system is the phase transition of the eigenvalue from the $\mathscr{PT}$-symmetric phase to the $\mathscr{PT}$-broken phase at a specific exceptional point, i.e. a $\mathscr{PT}$ breaking point (BP), where a pair of real eigenvalues become complex conjugate. In the $\mathscr{PT}$-symmetric phase of real eigenvalues, lots of wonderful phenomena analogous to those in conservative systems appear due to the strict loss-gain balance^28^ near the BP within the $\mathscr{PT}$-broken phase, the optical field localization effect playing a dominant role, leads to an energy accumulation and thereby some originally unimpressive effects, such as the nonlinearity induced by the light-matter interactions, can be gradually amplified. Generally, some system characteristics also may experience an abrupt phase transition near the BP. In view of this, $\mathscr{PT}$-symmetric systems have been widely used to optimize the desired effects, such as low-power optical isolation^29–32^, optomechanically induced transparency (OMIT)^21,33^, ultralow-threshold optical chaos^22^, single-mode microcavity laser or phonon lasing^23,34,35^, controllable optical responses^36,37^. Especially, a dimer system with balanced loss and gain, which is composed of a passive cavity and an active cavity directly coupled with each other, can show $\mathscr{PT}$-symmetric characteristics^30,34^.

Recently, the enhanced nonlinearity based on $\mathscr{PT}$ symmetry of an optomechanical system or a cavity system coupled with a quantum emitter (QE) has a great deal of researches^38–42^. Especially, the QE-cavity-coupling system, where cavities are directly coupled with each other, is an ideal platform for investigating nonlinear optics due to its performance advantages and easiness to be implemented^27^. Most recently, the trimer system involving three cavities has been used to study the phase transition and shown its excellent controllability^43^. Here we expect to build a novel QE-cavity-coupling trimer system where two cavities are indirectly coupled via a QE to achieve $\mathscr{PT}$ symmetry and investigate optical nonlinearity based on it. In view of this, a flexible and valid trimer system with two orthogonal cavities and a two-level QE is proposed in this paper. With an enough small spontaneous decay of the QE, the trimer system exhibits $\mathscr{PT}$-symmetric properties involving an approximate BP, around which both the linear and nonlinear parts of the transmission field can be significantly enhanced.

## Model and Methods

**Figure 1:**
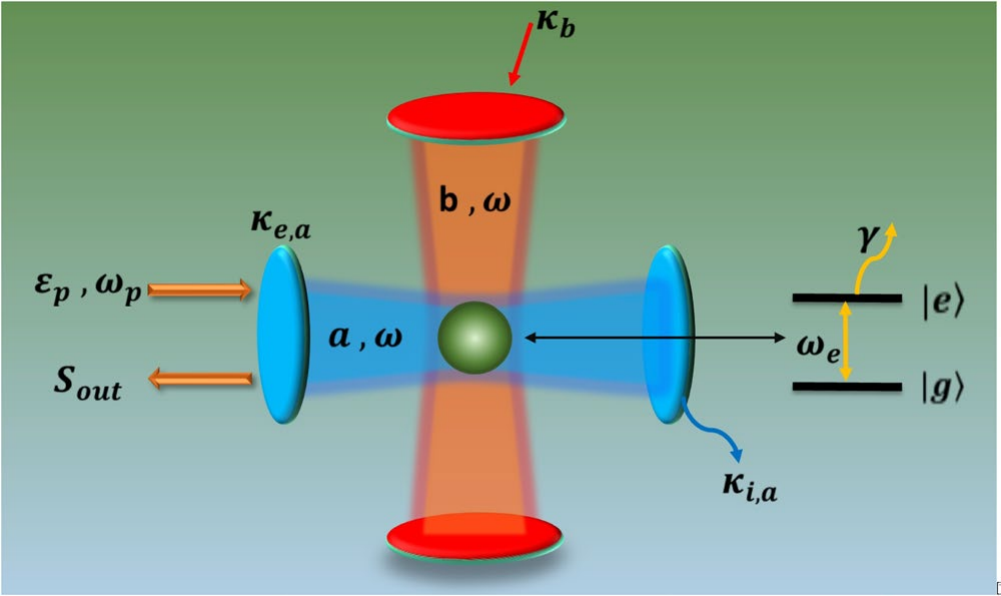
Schematic illustration of the $\mathscr{PT}$-symmetric trimer system including a horizontal cavity, a vertical cavity and a two-level QE. The horizontal cavity is coherently driven by a weak probe field with the amplitude *ε*_*p*_ and the frequency *ω*_*p*_. *S*_*out*_ denotes the output field of the horizontal cavity. The vertical cavity can become active due to an additional external pumping field.

We consider an optical trimer system made up of two standing-mode microresonators, i.e., Fabry-Pérot micro-cavities, which are not coupled directly due to their orthogonality with each other, and a two-level QE (or an atom) placed at the intersection of two cavities as shown in Fig. 1. The horizontal cavity of the resonance frequency *ω*_*a*_ is described by the annihilation (creation) operator *a* (*a*^†^); the vertical one of the resonance frequency *ω*_*b*_ is described by the annihilation (creation) operator *b* (*b*^†^), with assumption *ω*_*a*_ = *ω*_*b*_ = *ω*. The two-level QE has a transition between the ground state |*g*〉 and excited state |*e*〉 with frequency *ω*_*e*_, which can be driven by the intracavity fields. This transition process is described by the Pauli descending (ascending) operator *σ*_−_ = |*g*〉〈*e*| (*σ*_+_ = |*e*〉〈*g*|), obeying the commutation relation [*σ*_+_, *σ*_−_] = 2*σ*_*z*_. Two uncoupled cavities couple to the QE with the coupling strength *g*_*a*_ and *g*_*b*_, respectively. A monochromatic continuous-wave probe field of the amplitude *ε*_*p*_ and the frequency *ω*_*p*_ incident upon the horizontal cavity and coherently drive the cavity mode *a*. After the frame rotation with respect to the probe frequency, the Hamiltonian of this trimer system can be written as

1 $$\begin{array}{rcl} {\mathcal H}  & = & {\rm{\Delta }}({a}^{\dagger }a+{b}^{\dagger }b)+({\rm{\Delta }}+\delta ){\sigma }_{+}{\sigma }_{-}+i{g}_{a}(a{\sigma }_{+}-{a}^{\dagger }{\sigma }_{-})+i{g}_{b}(b{\sigma }_{+}-{b}^{\dagger }{\sigma }_{-})\\  &  & +\,i\sqrt{{\kappa }_{ae}}({\varepsilon }_{p}{a}^{\dagger }-H.c.)\end{array}$$

with detunings Δ = *ω* − *ω*_*p*_ and *δ* = *ω*_*e*_ − *ω* as well as the QE-cavity coupling strength ${g}_{a,b}={\mu }_{eg}\sqrt{\omega /2\hslash {\varepsilon }_{0}{V}_{a,b}}$ where *μ*_*eg*_ is the transition dipole moment of the QE and *V*_*a*,*b*_ is the mode volume of the cavity. We can assume *g*_*a*_ = *g*_*b*_ = *g* while assuming two cavities with appropriate parameters, such as the free length. The total loss of the horizontal cavity is described by *κ*_*a*_ = *κ*_*ai*_ + *κ*_*ae*_, where *κ*_*ai*_ is the intrinsic decay rate and *κ*_*ae*_ is the external loss rate due to the partial reflection of the driven mirror.

Phenomenologically including losses in the cavities and the QE, the semiclassical Heisenberg-Langevin equations of motion for the complicated trimer system can be written as

2 $$\begin{array}{rcl}\dot{a} & = & -(i{\rm{\Delta }}+\frac{{\kappa }_{ai}+{\kappa }_{ae}}{2})a-g{\sigma }_{-}+\sqrt{{\kappa }_{ae}}{\varepsilon }_{p}\\ \dot{b} & = & -(i{\rm{\Delta }}+\frac{{\kappa }_{b}}{2})b-g{\sigma }_{-}\\ {\dot{\sigma }}_{z} & = & -\gamma ({\sigma }_{z}+\frac{1}{2})+g(a{\sigma }_{-}^{\ast }+{a}^{\ast }{\sigma }_{-}+b{\sigma }_{-}^{\ast }+{b}^{\ast }{\sigma }_{-})\\ {\dot{\sigma }}_{-} & = & -[i({\rm{\Delta }}+\delta )+\frac{\gamma }{2}]{\sigma }_{-}-2g(a+b){\sigma }_{z}\end{array}$$

with the intrinsic decay rate *κ*_*b*_ of the vertical cavity and the spontaneous decay rate *γ* of the QE. Here, we assume *o* = 〈*o*〉 (*o* = *a*, *b*, *σ*_*z*_, *σ*_−_) in the semiclassical approximation within the weak-coupling regime (*g* < *κ*_*a*_/2)^40,44,45^.

A perturbation expansion method can be applied to obtain the steady-state solution of Eq. (2) in the weak-excitation approximation which is always tenable here due to the weak probe field. In fact, by performing an analytic calculation, a probe field with amplitude ${\varepsilon }_{p}\lesssim 0.01\sqrt{\gamma }$, which is reasonable in experiments, is low enough to guarantee the weak-excitation approximation with the other parameters as shown in this paper. The expanded form of the steady-state solution is given by

3 $$\begin{array}{rcl}a & = & \eta {a}^{(1)}+{\eta }^{2}{a}^{(2)}+{\eta }^{3}{a}^{(3)}+\mathrm{...}\\ b & = & \eta {b}^{(1)}+{\eta }^{2}{b}^{(2)}+{\eta }^{3}{b}^{(3)}+\mathrm{...}\\ {\sigma }_{z} & = & {\sigma }_{z}^{(0)}+\eta {\sigma }_{z}^{(1)}+{\eta }^{2}{\sigma }_{z}^{(2)}+{\eta }^{3}{\sigma }_{z}^{(3)}+\mathrm{...}\\ {\sigma }_{-} & = & {\sigma }_{-}^{(0)}+\eta {\sigma }_{-}^{(1)}+{\eta }^{2}{\sigma }_{-}^{(2)}+{\eta }^{3}{\sigma }_{-}^{(3)}+\mathrm{...}\end{array}$$

with a small continuous coefficient *η* ∈ [0, 1]. As the weak probe field results in the weak-excitation of the QE, the electrons mostly populate the ground state |*g*〉. Therefore, we could use ${\sigma }_{z}^{(0)}=-1/2$ and ${\sigma }_{-}^{(0)}=0$ for the zeroth-order steady-state solution^40,41^. With Eqs (2) and (3) and the expansions up to the third order of the given zeroth-order values, we can express the steady-state solution of *a* as

4 $${a}^{(1)}=\frac{\sqrt{{\kappa }_{ae}({f}_{1}{f}_{2}+{g}^{2})}}{{f}_{1}{f}_{2}{f}_{3}+{g}^{2}({f}_{2}+{f}_{3})}{\varepsilon }_{p}$$

5 $${a}^{(2)}=0$$

6 $$\begin{array}{rcl}{a}^{(3)} & = & \frac{2{g}^{4}{f}_{1}{f}_{2}}{\gamma [{f}_{1}{f}_{2}{f}_{3}+{g}^{2}({f}_{2}+{f}_{3})]}({f}_{1}+{f}_{1}^{\ast })\frac{{f}_{2}}{{f}_{1}{f}_{2}+{g}^{2}}\\  &  & \,\times {|\frac{{f}_{2}}{{f}_{1}{f}_{2}+{g}^{2}}|}^{2}{a}^{(1)}{|{a}^{(1)}|}^{2}\end{array}$$

where *f*_1_ = *γ*/2 + *i*(Δ + *δ*), *f*_2_ = *κ*_*b*_/2 + *i*Δ and *f*_3_ = (*κ*_*ai*_ + *κ*_*ae*_)/2 + *i*Δ. According to the standard input-output relation ${S}_{out}={S}_{in}-\sqrt{{\kappa }_{ae}}a$^46,47^, the transmission field of the horizontal cavity can be written as

7 $$\begin{array}{rcl}{S}_{out} & = & {\varepsilon }_{p}-\sqrt{{\kappa }_{ae}}{a}^{(1)}-\sqrt{{\kappa }_{ae}}{a}^{(3)}\\  & = & {t}_{a}^{(1)}{\varepsilon }_{p}-{t}_{a}^{(3)}{\varepsilon }_{p}{|{\varepsilon }_{p}|}^{2}\end{array}$$

with the first-order and the third-order transmission coefficient

8 $${t}_{a}^{(1)}=1-\frac{{\kappa }_{ae}({f}_{1}{f}_{2}+{g}^{2})}{{f}_{1}{f}_{2}{f}_{3}+{g}^{2}({f}_{2}+{f}_{3})}$$

9 $$\begin{array}{rcl}{t}_{a}^{(3)} & = & \frac{2{g}^{4}{\kappa }_{ae}^{2}{f}_{1}{f}_{2}}{\gamma [{f}_{1}{f}_{2}{f}_{3}+{g}^{2}({f}_{2}+{f}_{3})]}({f}_{1}+{f}_{1}^{\ast })\frac{{f}_{2}}{{f}_{1}{f}_{2}+{g}^{2}}\\  &  & \times {|\frac{{f}_{2}}{{f}_{1}{f}_{2}+{g}^{2}}|}^{2}\frac{{f}_{1}{f}_{2}+{g}^{2}}{{f}_{1}{f}_{2}{f}_{3}+{g}^{2}({f}_{2}+{f}_{3})}\\  &  & \times {|\frac{{f}_{1}{f}_{2}+{g}^{2}}{{f}_{1}{f}_{2}{f}_{3}+{g}^{2}({f}_{2}+{f}_{3})}|}^{2}\end{array}$$

It is worth to note that ${T}_{a}=|{t}_{a}^{(1)}{|}^{2}$ and ${K}_{a}={Re}[{t}_{a}^{(3)}]$ can describe the linear transmission rate and the nonlinear intensity of the transmission field, respectively. Obviously, the nonlinear intensity strongly depends on the cavity-QE-coupling strength *g*. If setting *g* = 0, this trimer system is reduced to a single-cavity system, thereby *K*_*a*_ = 0 means that nonlinear nature disappears in the absence of the QE.

## Results and Discussion

### $\mathscr{PT}$ symmetry characteristics

This double-cavity trimer system can be transformed between the passive-passive scheme (*κ*_*a*_ > 0, *κ*_*b*_ < 0) and passive-active scheme (*κ*_*a*_ > 0, *κ*_*b*_ < 0) by precisely adjusting the additional external pumping field on the vertical cavity^39,48–50^. Now we consider that *κ*_*a*_ = −*κ*_*b*_ = *κ* and the decay rate of the QE satisfies $\gamma \ll \kappa$, we can safely neglect the QE loss (*γ* = 0) temporarily, then the matrix form of the Hamiltonian can be written as

10 $${\bf{H}}=(\begin{array}{ccc}{\rm{\Delta }}-i\frac{\kappa }{2} & g & 0\\ g & {\rm{\Delta }} & g\\ 0 & g & {\rm{\Delta }}+i\frac{\kappa }{2}\end{array})$$

here, for simplicity, we assume that the transition of the two-level QE is resonant with the modes of two cavities, i.e. *δ* = 0. Then the eigenvalues of the Hamiltonian can be obtained as

11 $$\begin{array}{rcl}{\lambda }_{0} & = & {\rm{\Delta }}\\ {\lambda }_{\pm } & = & {\rm{\Delta }}\pm \sqrt{2{g}^{2}-\frac{{\kappa }^{2}}{4}}\end{array}$$

**Figure 2:**
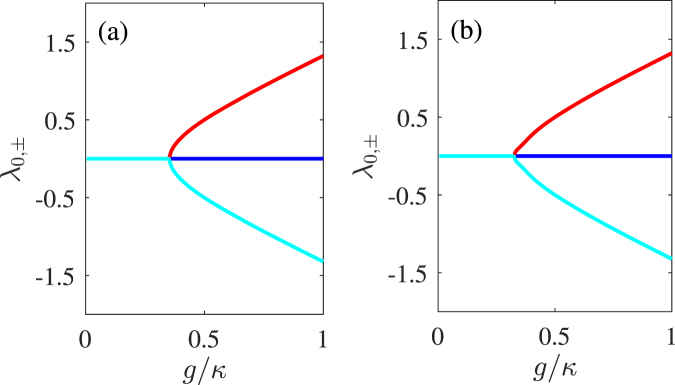
The bifurcation diagrams of the eigenvalues *λ*_0_ (blue), *λ*_+_ (red) and *λ*_−_ (celeste) of the $\mathscr{PT}$-symmetric trimer system Hamiltonian versus the cavity-QE-coupling strength *g* with *γ* = 0 in (**a**) and with *γ* = 0.025*κ* in (**b**). We set *κ*_*a*_ = − *κ*_*b*_ = *κ* as a unit, other parameters *κ*_*ai*_ = 0.25*κ*, *κ*_*ae*_ = 0.75*κ* and Δ = *δ* = 0 are chosen according to the recent experimental parameters^34^.

Obviously, the one of three eigenvalues *λ*_0_ can maintain real and then satisfy the $\mathscr{PT}$ symmetry^43^ the other two eigenvalues *λ*_±_ undergo $\mathscr{PT}$ phase transitions near the $g=\kappa /2\sqrt{2}$, similar to the case in a typical dimer system, which comprises just one passive and one active cavities with balanced loss and gain coupled directly with each other^23,30,31^. That means our trimer system can provide an alternative platform for optical $\mathscr{PT}$ symmetry operation. In this system, the passive and active cavities with balanced loss and gain are coupled indirectly with each other, but both couple directly the QE. As the QE builds an interaction bridge for two cavities, the trimer system shows the similar $\mathscr{PT}$-symmetric characteristics as the dimer one now. Moreover, this trimer proposal may be used to study $\mathscr{PT}$-symmetric nonlinearity, and provide more controllable parameters due to introducing a QE. Relative characteristics can be directly observed in Fig. 2(a), where we plot the analytical results of the $\mathscr{PT}$-symmetric structure according to Eq. (11). Then, we could consider the case of $g > \kappa /2\sqrt{2}$ as the $\mathscr{PT}$-symmetric phase regime and the case of $g < \kappa /2\sqrt{2}$ as the $\mathscr{PT}$-broken phase regime, respectively.

In Fig. 2(b), we also plot the numerical results of the actual situation by taking into account a nonvanishing value of the spontaneous decay rate of the QE (*γ* = 0.025*κ*)^34^. Whether in panel (a) or (b), it represents the $\mathscr{PT}$-symmetric characteristics that *λ*_+_ and *λ*_−_ are symmetrical and centered on *λ*_0_. In addition, Fig. 2(b) shows the transformation between the $\mathscr{PT}$-symmetric phase and $\mathscr{PT}$-broken phase around the BP, which is analogous to the one in Fig. 2(a). It can be found that the existence of *γ* just leads to a tiny shift of the BP position and a slight reduction of the change rate of the phase transition around BP, rather than destroying the $\mathscr{PT}$ symmetry. That means, even though taking into account a reasonable value of *γ*, we could also achieve $\mathscr{PT}$ symmetry involving a phase transition. Then, we further explore the applications of this $\mathscr{PT}$-symmetric proposal for nonlinear optics in next section.

### *Enhanced nonlinear characteristics*

**Figure 3:**
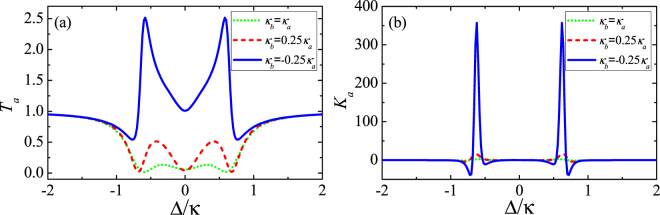
The linear transmission rate *T*_*a*_ (**a**) and the third-order nonlinear intensity *K*_*a*_ (**b**) as functions of the detuning Δ with *κ*_*b*_ = *κ*_*a*_ (green-dotted line); *κ*_*b*_ = 0.25*κ*_*a*_ (red-dashed line) and *κ*_*b*_ = −0.25*κ*_*a*_ (blue-solid line). We set *κ*_*a*_ = *κ*, *γ* = 0.025*κ* and *g* = 0.45*κ*. Other parameters are the same as those in Fig. 2.

Figure 3 shows the linear transmission rate *T*_*a*_ and the third-order Kerr nonlinear intensity *K*_*a*_ as functions of the detuning Δ with different values of *κ*_*b*_. In Fig. 3(a), when *κ*_*b*_ = *κ*_*a*_ = *κ* corresponding to a passive-passive system, two symmetric perfect absorption windows and an on-resonance dip appear; when *κ*_*b*_ = 0.25*κ* corresponding to a reduction of the vertical cavity loss, it shows somewhat increasing linear output intensity, i.e., two symmetric peaks become obvious, but the system is still in an electromagnetically induced-absorption (EIA) regime; when *κ*_*b*_ = −0.25*κ* corresponding to the presence of gain effect within the vertical cavity, forming a passive-active system, the amplification of the linear output intensity emerges, which is featured by two amplified sideband peaks. In addition, a controllable optical switch between the tunable perfect absorption and the flexible enhanced linear output intensity can be achieved. As shown in Fig. 3(b), when the trimer system enters into the passive-active scheme, the amplified sideband peaks of the nonlinear output intensity are far higher than those in the passive-passive scheme. Note, although both linear and nonlinear parts of the transmission field are largely enhanced, the nonlinear part exhibits much more remarkable enhancement than the linear part, from a originally poor value, meaning that the proportion of the nonlinear part gets gradually increased and the trimer system becomes more and more nonlinear. In view of this, in this trimer system, the nonlinearity of the transmission field is enhanced with the assistance of the gain effect of the vertical active cavity.

**Figure 4:**
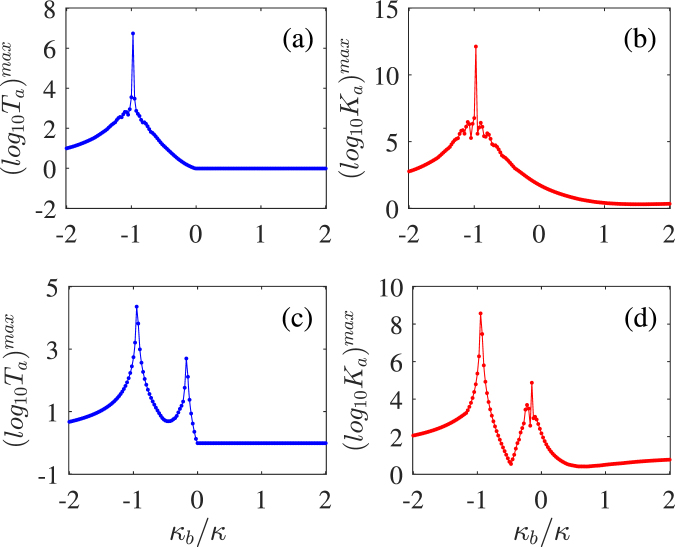
The maximal logarithms of the linear transmission rate *T*_*a*_ (**a**,**c**) and the third-order nonlinear intensity *K*_*a*_ (**b**,**d**) versus the intrinsic decay rate *κ*_*b*_ for *g* = 0.45*κ* (**a**,**b**) and *g* = 0.2*κ* (**c**,**d**). Other parameters are the same as those in Figs 2 and 3.

In order to further understand the role of the $\mathscr{PT}$-symmetric nature to the transmission field within the trimer system, we plot in Fig. 4 the maximal logarithms of the linear transmission rate *T*_*a*_ and the third-order nonlinear intensity *K*_*a*_ versus the intrinsic decay rare *κ*_*b*_ of the vertical cavity. As shown in Fig. 4(a) and (b), *T*_*a*_ and *K*_*a*_ begin to ascend quickly when the trimer system enters the passive-active regime of *κ*_*b*_ < 0 and soon develop into a sharp peak close to the point of *κ*_*b*_ = −*κ*, at which a $\mathscr{PT}$-symmetric structure is realized with balanced gain and loss. Then, *T*_*a*_ and *K*_*a*_ descend quickly when *κ*_*b*_ is decreased further beyond the $\mathscr{PT}$-symmetric point of *κ*_*b*_ = −*κ* but both maintain higher values than those in the passive-passive regime of *κ*_*b*_ > 0. The underlying physics for this effect seems that the trimer system enters the weak coupling regime and thus tends to be decoupled, when the $\mathscr{PT}$-symmetric point of *κ*_*b*_ = −*κ* is near the boundary of |*κ*_*b*_| = 2*g* = 0.9*κ* between the weak and strong coupling regimes. Figure 4(c) and (d) show however that *T*_*a*_ and *K*_*a*_ have two sharp peaks, with the left (right) one lying in the weak (strong) coupling regime of |*κ*_*b*_| > 2*g*(|*κ*_*b*_| < 2*g*)^40^, when the $\mathscr{PT}$-symmetric point of *κ*_*b*_ = −*κ* is far away from the boundary of |*κ*_*b*_| = 2*g* = 0.4*κ* between the weak and strong coupling regimes. The decrease of *T*_*a*_ and *K*_*a*_ for larger absolute values of *κ*_*b*_ near the right peak can be attributed once again to the fact that the trimer system is entering the weak coupling regime. But the left peak should be more relevant to the $\mathscr{PT}$-symmetric nature because it is always close to the $\mathscr{PT}$-symmetric point, i.e. insensitive to the coupling strength *g*. In view of this, both the linear and nonlinear parts of the transmission field can be significantly enhanced when the trimer system exhibits the $\mathscr{PT}$-symmetric nature no matter it works in the weak coupling regime or in the strong coupling regime.

**Figure 5:**
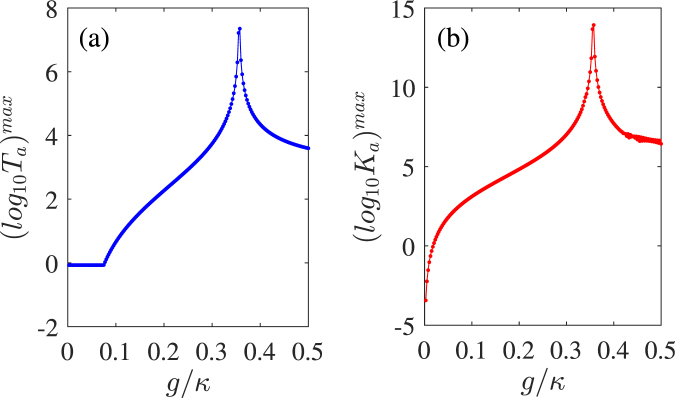
The maximal logarithms of the linear transmission rate *T*_*a*_ (**a**) and the third-order nonlinear intensity *K*_*a*_ (**b**) versus the QE-cavity coupling strength *g* in the $\mathscr{PT}$-symmetric case of *κ*_*b*_ = −*κ*. Other parameters are the same as those in Figs 2 and 3.

As analytically predicted above, the $\mathscr{PT}$-phase transition should occur near the BP where $g=\kappa /2\sqrt{2}\simeq 0.354\kappa$^43^. Then, we examine the behaviors of the linear part *T*_*a*_ and the nonlinear part *K*_*a*_ of the transmission field near the BP. In Fig. 5, we plot the maximal logarithms of *T*_*a*_ and *K*_*a*_ as functions of the QE-cavity-coupling strength *g*/*κ*. Here we change *g*/*κ* ∈ [0, 0.5] for satisfying the weak-coupling regime^40^. In Fig. 5, when *g* is very small, i.e. the QE-cavity coupling is extremely weak, which leads to a decoupled system located in the EIA regime. With *g* gradually increasing within the $\mathscr{PT}$-broken phase $(g < \kappa /2\sqrt{2})$ of the trimer system, the strong mutual coupling supports a compensation for the horizontal passive cavity loss and then *T*_*a*_ increases exponentially until the BP. *T*_*a*_ reaches the maximal value near the BP featured by a narrow sharp peak. Once *g* increases beyond the BP within the $\mathscr{PT}$-symmetric phase, the enhanced *T*_*a*_ may rapidly reduce. A similar behavior of the enhanced nonlinear parts can be observed in Fig. 5(b), and achieve a maximal value around the BP as well. Therefore, it is feasible to further enhance both the linear and the nonlinear parts of the transmission field by adjusting *g* approaching to the BP within the $\mathscr{PT}$-broken phase. Physically, in the $\mathscr{PT}$-broken phase, the field localization effect can leads to the dynamical accumulations of the intracavity energy, which will reach the acme near the BP^2,38,40^. Hence, when *g* is big enough, the originally weak linear part arising from the puny probe driving and the weak nonlinear part induced by the QE-cavity coupling are both gradually amplified and reach the maximal values near the BP. In view of this, the nonlinear characteristics of the system get enhanced. However, with the further increasing of *g*, a strong tunneling effect becomes dominant to suppress the localization effect, so that *T*_*a*_ and *K*_*a*_ will decrease rapidly and reduce to a stable level due to the QE saturation^41^.

**Figure 6:**
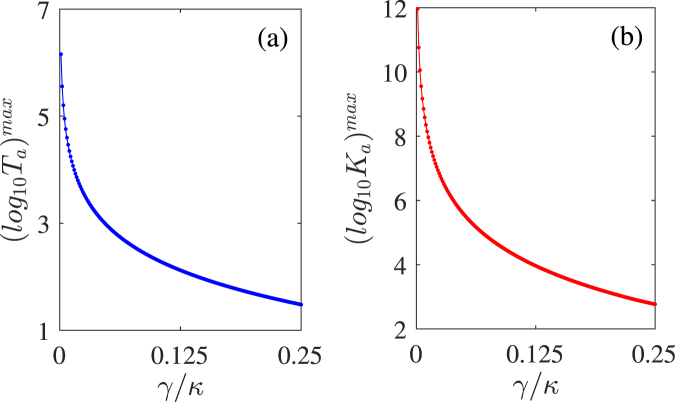
The maximal logarithms of *T*_*a*_ (**a**) and *K*_*a*_ (**b**) versus the QE spontaneous decay rate *γ* in the $\mathscr{PT}$-symmetric case of *κ*_*b*_ = −*κ* and *g* = 0.45*κ*. Other parameters are the same as for Figs 2 and 3.

Up to now, we have always used a reasonable and actual value *γ*/*κ* = 0.025, which is much smaller than the double-cavity loss, to maintain an satisfactory $\mathscr{PT}$ symmetry. As showed in Fig. 2, this trimer system may show more ideal $\mathscr{PT}$ symmetry with *γ*/*κ* = 0, and its $\mathscr{PT}$-symmetric nature will gradually weaken with the increasing *γ*. Thus it is necessary to examine the enhanced *T*_*a*_ and *K*_*a*_ with varying *γ* in order to discuss the relation between the gain degree of the linear and nonlinear parts of the transmission field and the spontaneous decay rate of the QE. In Fig. 6, we plot the maximal logarithms of *T*_*a*_ and *K*_*a*_ as functions of *γ*/*κ* ∈ [0, 0.25]. Figure 6 shows a significant negative correlation between *γ* and *T*_*a*_ (*K*_*a*_) which reaches its maximum with *γ*/*κ* = 0. The results are in line with our expectation, which is the enhanced nonlinearity should be derived from the $\mathscr{PT}$ symmetry of this trimer system. The physical reason why the linear and nonlinear parts of the transmission field reduce with the increasing of *γ* is that the strong spontaneous decay of the QE will break the loss-gain balance, which is the precondition for $\mathscr{PT}$ symmetry. In addition, as shown in Fig. 6, both *T*_*a*_ and *K*_*a*_ could still stay at a satisfactory level (~30 and ~144, respectively) even though *γ* is considerable compared with the intrinsic decay rate *κ*_*ai*_.

## Conclusions

In summary, we have proposed a trimer system composed of two uncoupled passive and active cavities and a two-level QE building an indirect interaction bridge for two cavities. Under the loss-gain balance of two cavities, the results show that, when the QE spontaneous decay rate *γ* = 0, the trimer system will show a strict $\mathscr{PT}$ symmetry; when *γ* has an actual value and is small enough compared with the total loss of the cavity, the $\mathscr{PT}$-symmetric characteristics will weaken but still maintain the $\mathscr{PT}$-broken point (BP). One of excellent applications of this $\mathscr{PT}$-symmetric trimer system is the enhanced transmission field and nonlinear characteristics, i.e. both the linear and nonlinear parts of the transmission field can be significantly enhanced, and the proportion of the nonlinear part gets improved. Even though in the unbalanced loss-gain case, the linear and nonlinear transmission parts can also be enhanced to a satisfactory degree in this trimer system. One can further maximize the linear and nonlinear transmission parts by adjusting the QE-cavity coupling strength, which is in order to set the system around the BP. In addition, there is a significant negative correlation between the gain degree of the linear (nonlinear) transmission part and the QE spontaneous decay, because the increasing of *γ* will break the balanced loss-gain condition of $\mathscr{PT}$ symmetry. This flexible and novel trimer proposal of ideal $\mathscr{PT}$ symmetry with more tuned parameters is feasible for experiments and may open up a new path to investigating the $\mathscr{PT}$-symmetric optics of the low-light level. And the enhanced nonlinearity induced by this novel QE-cavity coupling may provide a promising platform of controllable optical responses for fabricating photonic devices and controllable nonlinear optical media for quantum information and communication of photons.
